# Supplementary material for: Orally Administered Exosomes Suppress Mouse Delayed-Type Hypersensitivity by Delivering miRNA-150 to Antigen-Primed Macrophage APC Targeted by Exosome-Surface Anti-Peptide Antibody Light Chains
Source: Int J Mol Sci. 2020 Aug 2;21(15):5540. doi: 10.3390/ijms21155540 (PMC7432818; doi:10.3390/ijms21155540)
Supplement: Supplementary file 1 [file ijms-21-05540-s001.pdf]

Orally Administered Exosomes Suppress Mouse Delayed-Type Hypersensitivity by Delivering miRNA-150 to Antigen-Primed Macrophage APC Targeted by Exosome-Surface Anti-Peptide Antibody Light Chains

Katarzyna Nazimek, Krzysztof Bryniarski, Wlodzimierz Ptak, Tom Groot Kormelink and Philip W. Askenase

***Int. J. Mol. Sci.* 2020, 21(15), 5540; https://doi.org/10.3390/ijms21155540**

Supplementary figures S1 and S2

Figure S1.

The general scheme of Ag immunization and challenge testing of mice administered ID with ovalbumin (OVA) without an adjuvant to induce DTH responses.

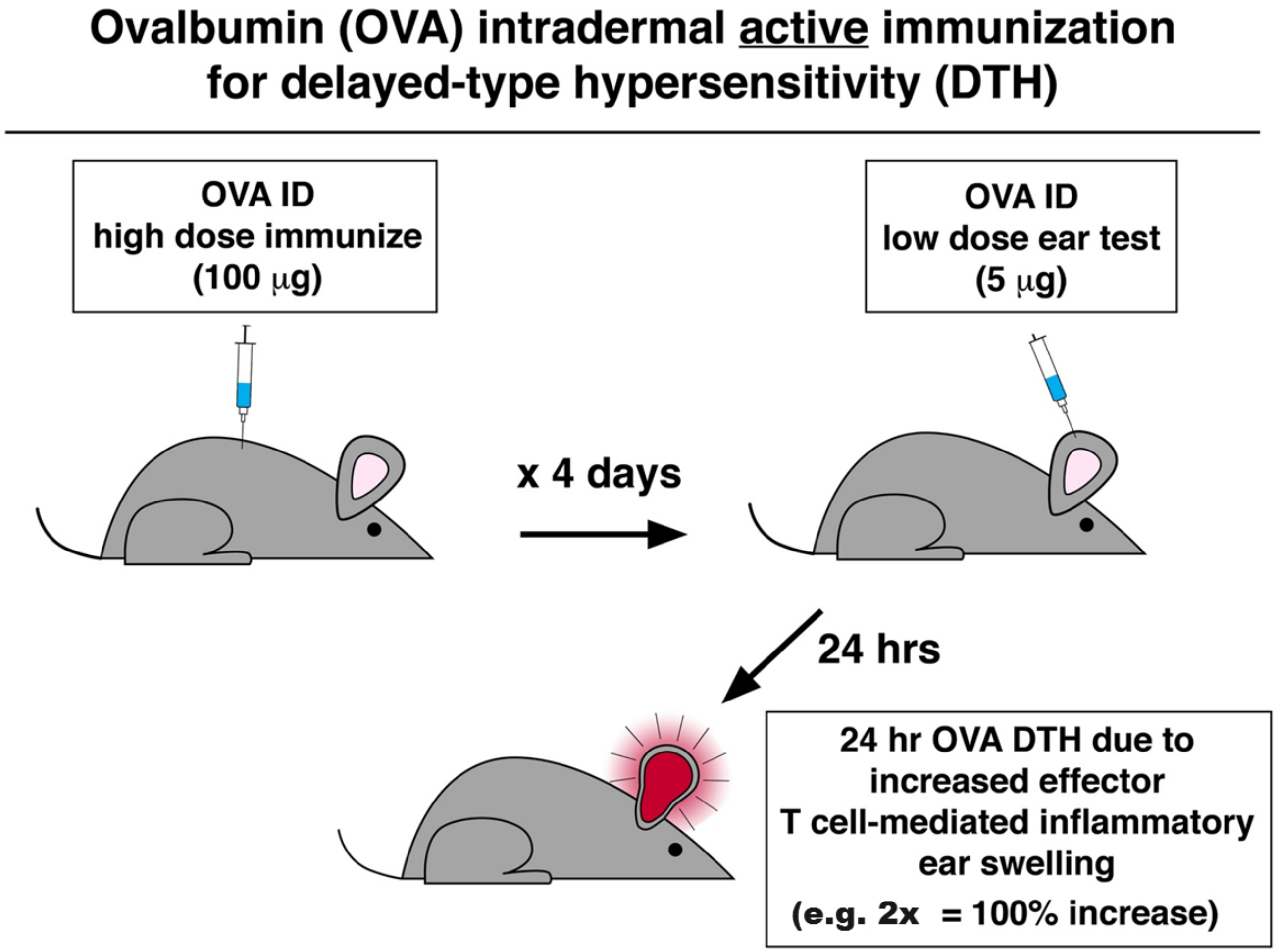

(S1A) Protocol for ID immunization and eliciting ear swelling responses after inducing OVA Ag DTH. CBA male mice were immunized ID twice with OVA in saline injected into four different sites of the abdomen in 50μL each, for a total dose of 100μg OVA. Four days later they were skin challenged ID in the ears with 5μg OVA and the subsequent ear swelling was measured with an engineer's micrometer at 2 and 24 hours, and, where indicated, daily up to 120 hours after challenge, by a well experienced reader, unaware of their experimental status. The data were recorded by an assistant who randomly selected mice for measurements and administered ether inhalation anesthesia for the ear readings. All readings were done in duplicate for each of both ears to obtain the single mean ear swelling determination for that mouse, at that single time point. Mice were kept fed and watered in a bacteriology hood in the laboratory for the duration of the experiment to protect them from infection and from the biological changes that might happen during transit to and from the animal quarters far away.

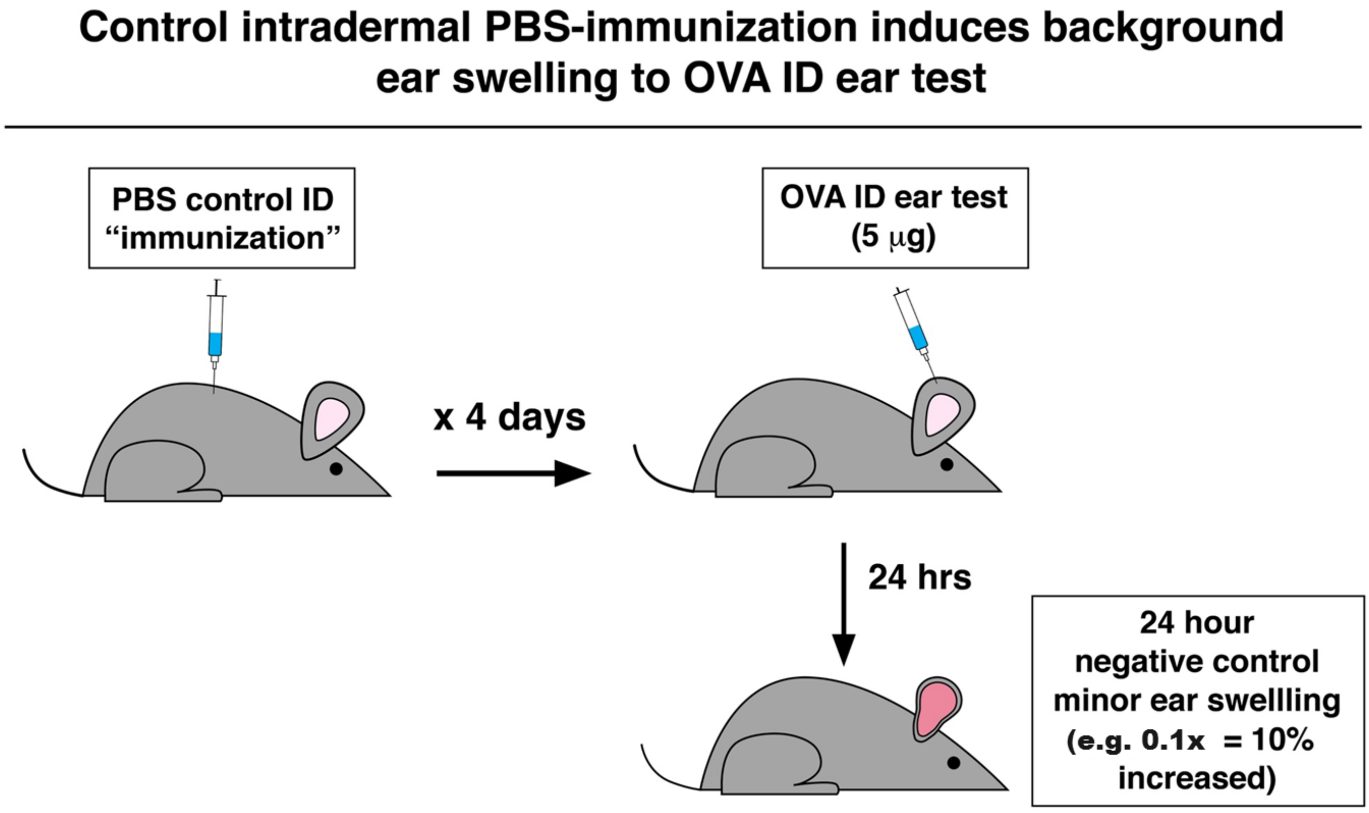

(S1B) Protocol for control ID immunization and OVA skin testing to verify OVA DTH. Immunization is as in **Figure S1A**, but with ID saline alone. Then, four days later, ear challenge was exactly as above with 5μg OVA ID. Ear thickness measurements were exactly as above in **Figure S1A**.

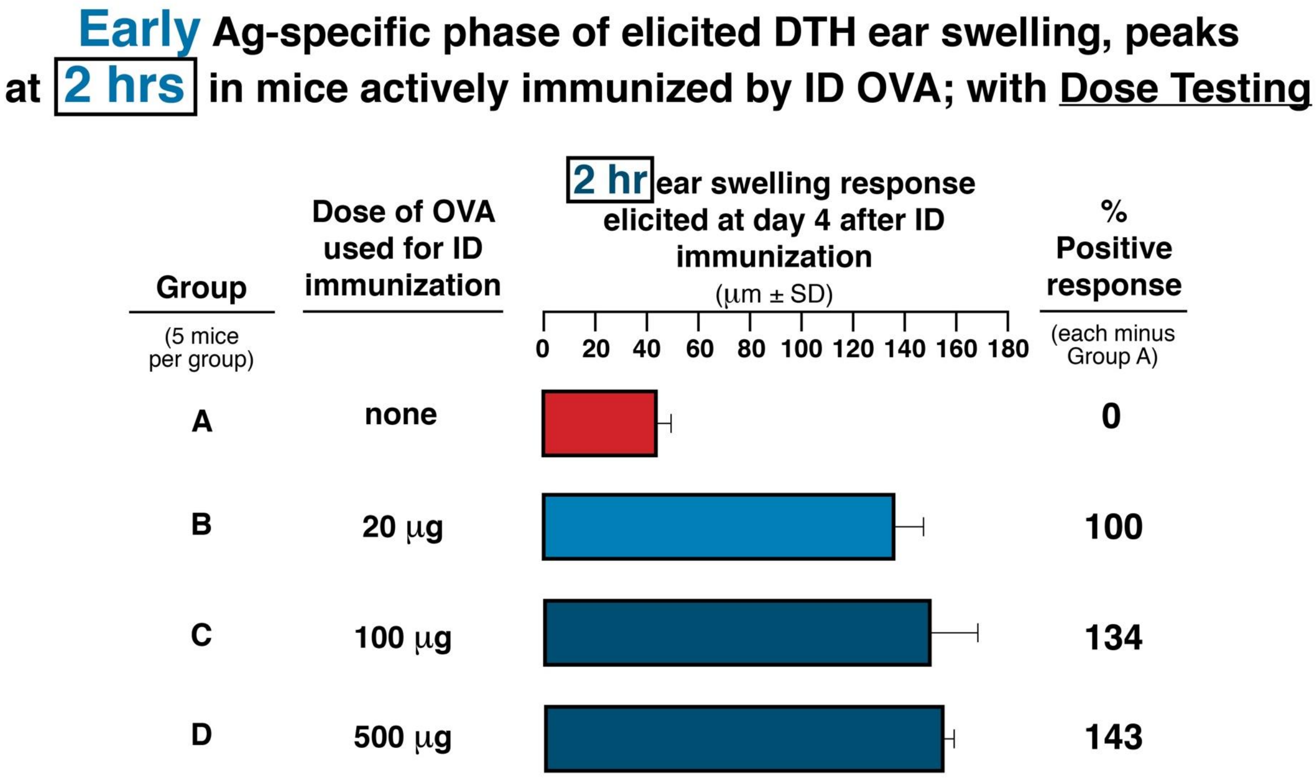

(S1C) Weakly Ag-specific early phase of DTH is elicited as ear swelling that peaks at 2 hours after challenge with ID injection of OVA into the ears of mice actively immunized ID on day 0 and 1 with OVA; performed here with immunizing dose response testing. Immunization was as in **Figure S1A**, with ID total immunizing doses in each separate mouse were 20 µg, vs. 100 µg, vs. 500 µg OVA at four ID sites each. Then at day four, the 5 µg OVA ID elicited ear swelling responses were tested at 2 hours to evaluate an early phase of DTH.

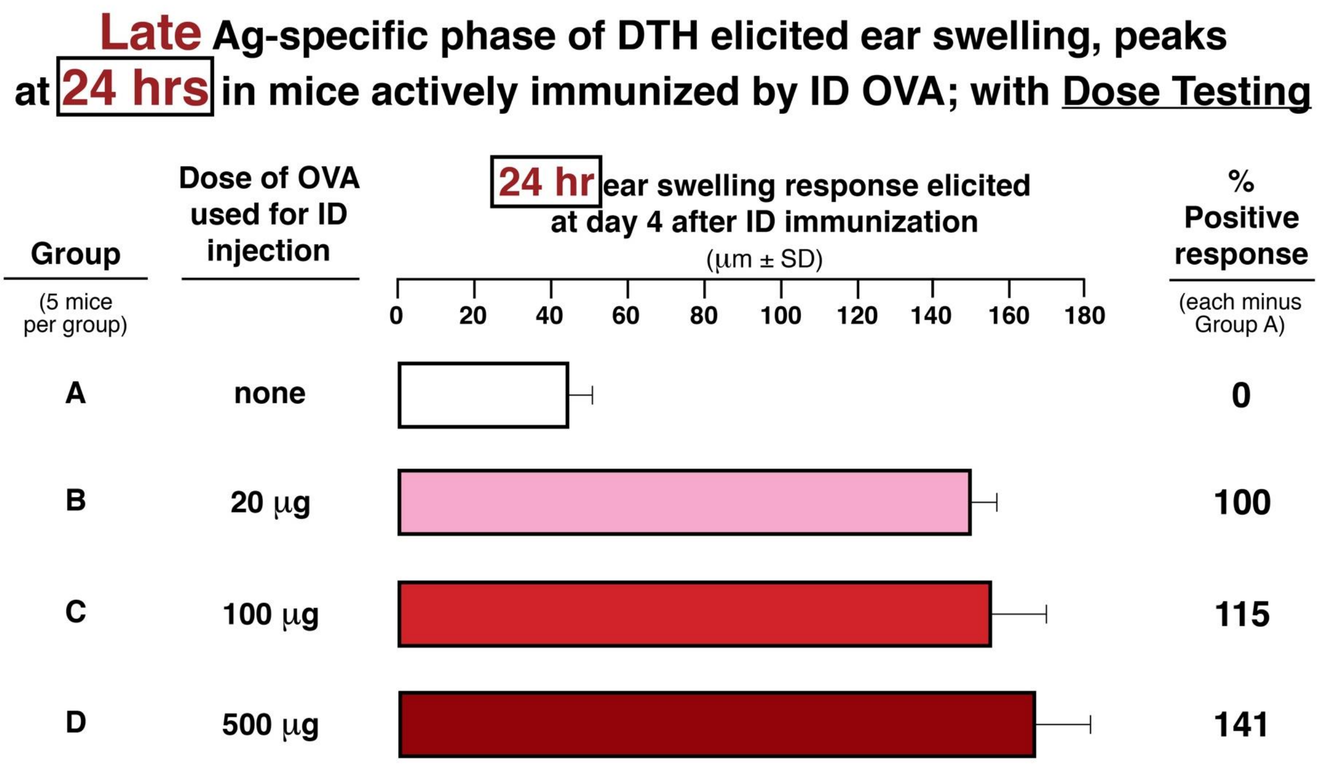

(S1D) <u>Late Ag-specific phase of DTH- elicited ear swelling response to local ID OVA injection, peaks at 24 hours in mice actively immunized ID with OVA; performed here with immunizing dose response testing.</u> The ear swellings at 24 hour after challenge testing of mice from **Figure S1C**.

Figure S2.

DTH induced by ID immunization with OVA is prevented by prior IV injection of high doses of OVA-linked autologous RBC that induce tolerance to OVA, due to suppressor T cells producing exosomes able to inhibit adoptive cell transfer of OVA-immune DTH.

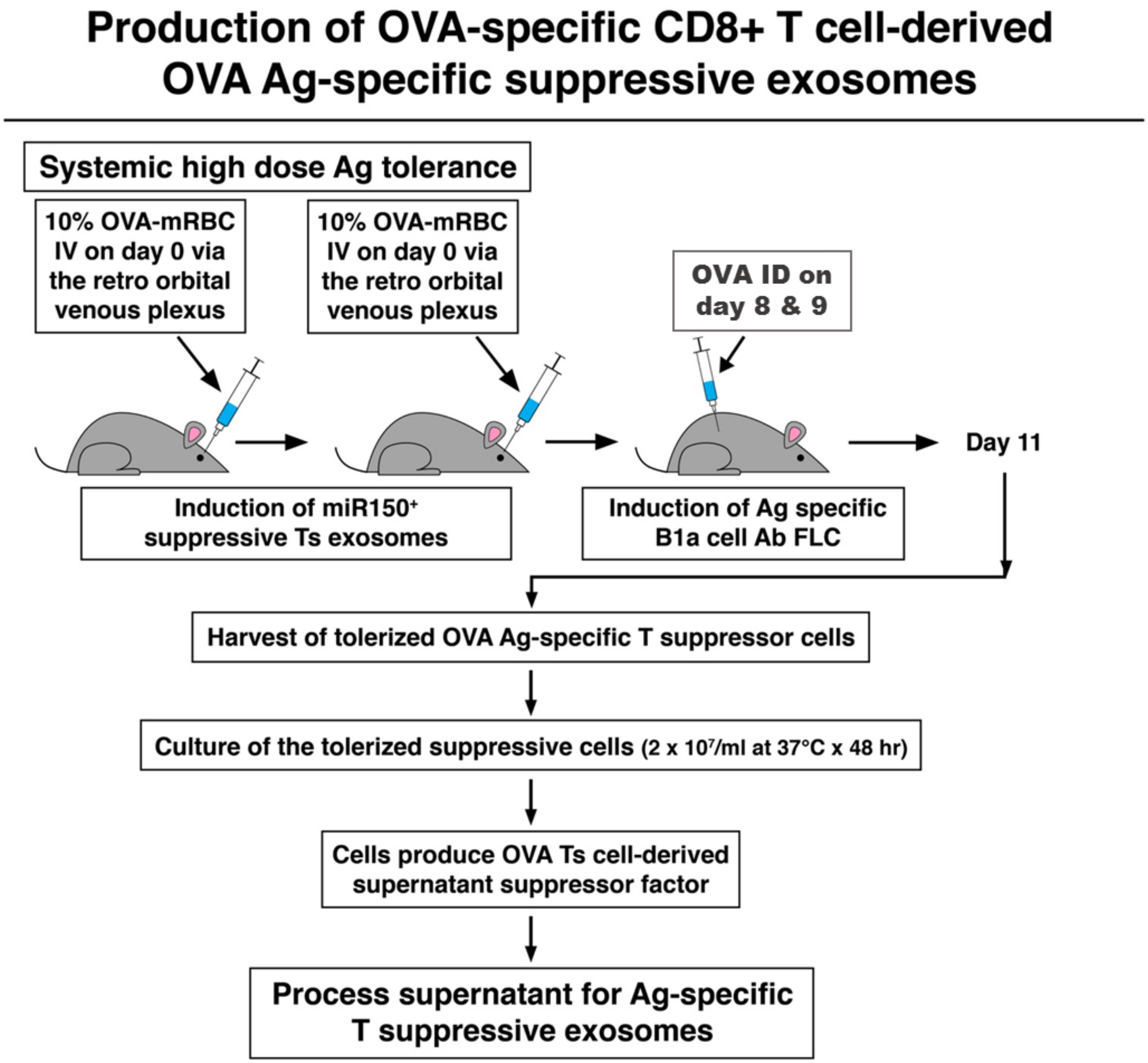

(S2A) Protocol for induction of OVA Ag-specific suppressor T cells and their suppressive exosomes. To induce immunological tolerance, mice were injected IV with a high dose of 10% autologous RBC-linked with OVA on day 0 and day 4. Then, on day 9, these mice received ID injections of OVA alone, as in **Figure S1A**. Then, on day 11, harvested lymph node and spleen cells were shown to contain suppressor T cells. The cells then were cultured in vitro at 37°C in protein free media at high density (2x10^7/ml) for 48 hours and then the resulting suppressive supernatant was processed to obtain the contained exosomes, i.e. the supernatant was cleared of debris and cells by light centrifugations ending at 3,000g, then was ultrafiltered down to 0.2 μm (200 nano Meter) filters and subsequently 4°C ultracentrifuged twice at 100,000g to obtain a pellet rich in the exosomes (**Figure S1A)**.

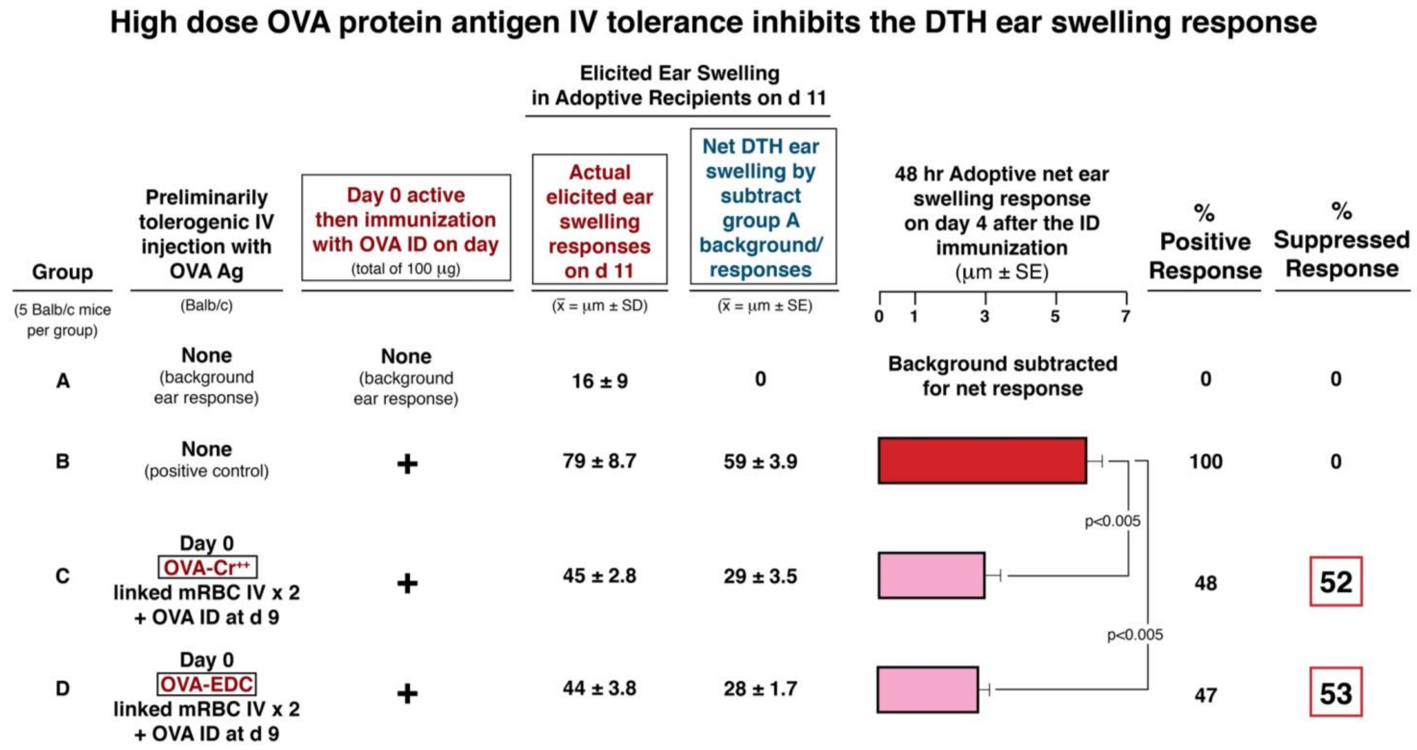

(S2B) DTH ear swelling 4 days after ID immunization with OVA in mice pretreated with OVA-linked to autologous RBC by different reagents. Mice were injected IV twice with 10% autologous RBC as above, after linkage of the RBC to OVA by chromium or EDC (Groups C and D). Then on day 9 these mice, and previously non injected positive controls (Group B) were ID immunized with OVA alone as in **Figure S1A**. Four days later all groups, and an additional group of totally naive background mice (Group A) were ear tested as above, with 5 μg OVA ID and elicited DTH ear swelling responses were measured.

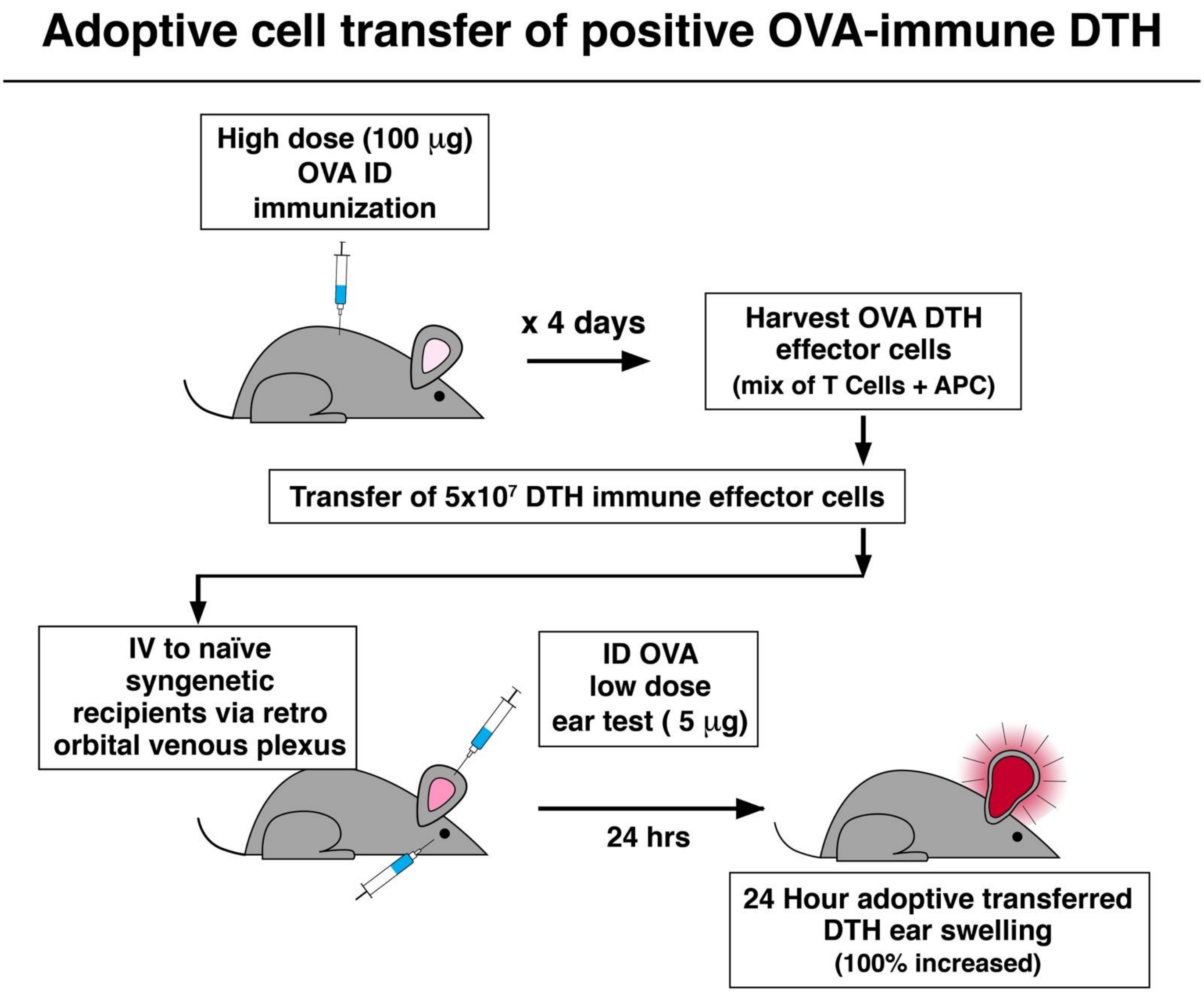

(S2C) Protocol for adoptive cell transfer of OVA-immune effector cells from ID skin immunized donors to elicit DTH responses in recipients. Cell donor mice were ID immunized twice with 100 μg OVA in saline, as in **Figure S1A**. On day 4 lymph node and spleen cells containing DTH-effector cells were harvested. Then their single-cell suspensions were washed and subsequently resuspended in a volume of 0.25 ml/eventual recipient that was injected IV into naive mice. A day later the recipients were ear tested with OVA injected ID to elicit DTH ear swelling, that was measured in the recipients 24 hours later.

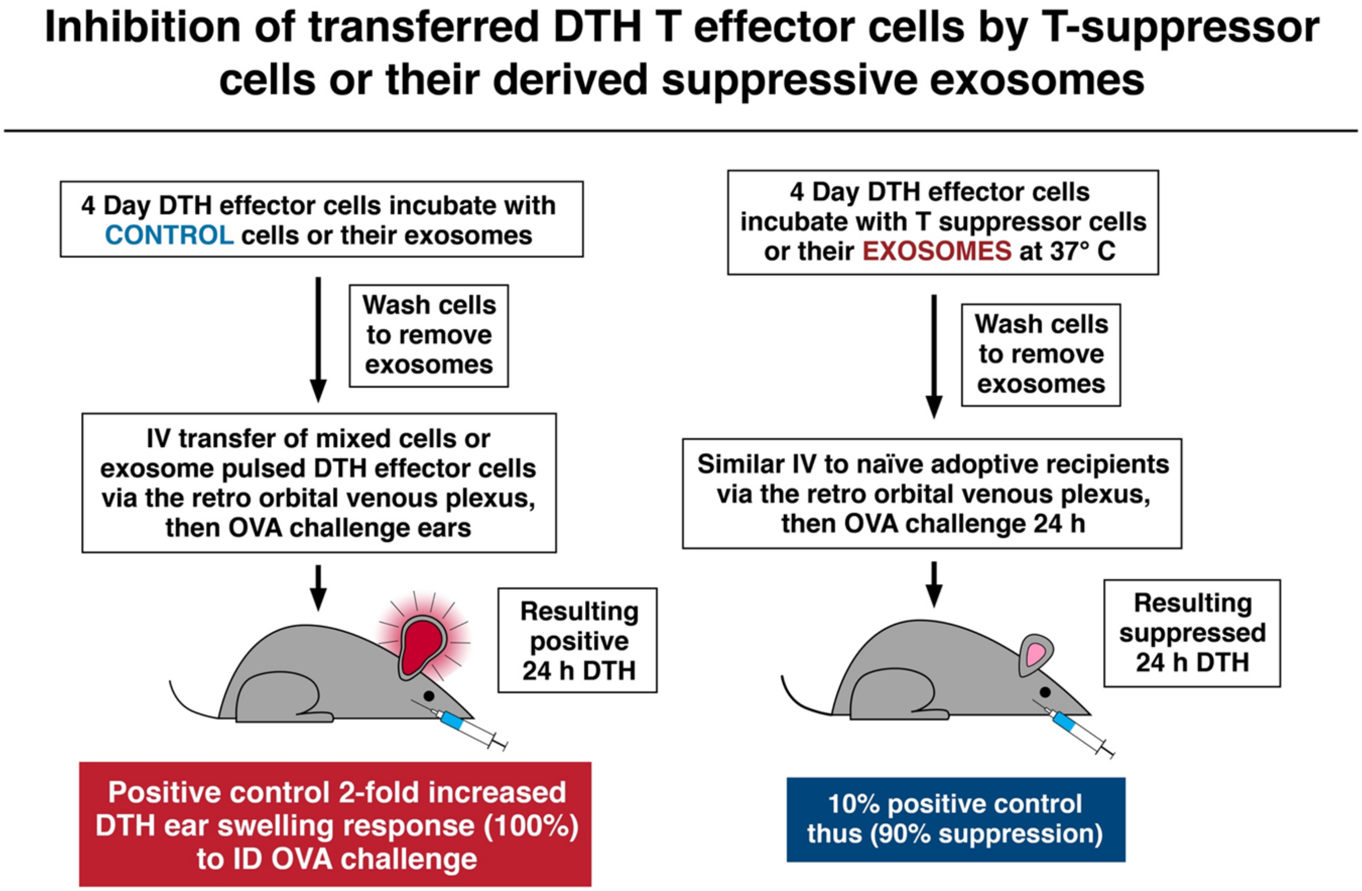

(S2D) Protocol for inhibition of adoptively transferred DTH-effector cells by suppressor T cells derived from OVA-tolerized mice, or their derived suppressive exosomes. Adoptive transfer of DTH-effector cells was performed as above. However in these cases, the harvested effector cells were incubated in vitro at 37°C for 30 minutes with the suppressor T cell-derived exosomes, at a ratio of 10^{10} nanovesicles per $7x10^7$ effector cells to be transferred per eventual recipient. Then, after washing, the cells freed of the exosomes by centrifugation at 300g were transferred IV. The control transfers are shown on the left, and the experimental groups on the right of the figure.
